# Arthroscopic Arthrolysis of a Completely Ankylosed Knee in Extension via a Superolateral Portal-First Approach

**DOI:** 10.7759/cureus.113783

**Published:** 2026-08-01

**Authors:** Long Luong Sinh, Nam Vu Tu, Nam Ta Van Thanh, Anh Ta Tuan

**Affiliations:** 1 College of Health Sciences, VinUniversity, Hanoi, VNM; 2 Orthopaedics, Vinmec International Hospital, Hanoi, VNM

**Keywords:** arthrofibrosis, arthroscopic arthrolysis, knee ankylosis, lysis of adhesions, superolateral portal

## Abstract

Complete knee ankylosis in full extension following fracture surgery poses a distinct technical challenge for arthroscopic intervention, because standard portal placement requires at least partial knee flexion to open the tibiofemoral joint space. We report a 61-year-old woman who developed complete left knee ankylosis at 0° three months after open reduction and internal fixation with bone grafting of the proximal tibia at an outside institution. Physical examination revealed a completely rigid knee with no passive or active flexion and severely restricted patellar mobility, and conservative management including physical therapy had failed. We employed a superolateral portal-first approach with the knee in full extension, targeting the preserved suprapatellar potential space beneath the superior patellar pole. Through systematic proximal-to-distal arthroscopic debridement of dense intra-articular adhesions involving the suprapatellar pouch, peripatellar gutters, infrapatellar region, and intercondylar notch, followed by gentle incremental manipulation, intraoperative flexion of 90° was achieved. At discharge on postoperative day 8, the patient had active flexion of 50°, passive flexion of 85° on continuous passive motion, and full extension. At one-month follow-up, she maintained full active extension with no extensor lag and had achieved active flexion of 90° by goniometry, and no complications occurred. No validated patient-reported outcome measure was recorded. The suprapatellar space typically retains a residual potential space even in severely ankylosed joints and therefore provides a reliable initial arthroscopic entry point; this proximal-to-distal strategy with progressive portal creation enables access without the need for initial knee flexion. A superolateral portal-first technique may represent a useful option for obtaining initial arthroscopic access in selected cases of complete post-traumatic knee ankylosis. A single case observed for one month cannot establish clinical success or durability, and no claim of superiority over conventional anterior portal techniques is made.

## Introduction

Arthrofibrosis of the knee is a fibrotic joint disorder that begins with an inflammatory reaction to insults such as injury, surgery, or infection, in which excessive extracellular matrix and adhesions contract the joint recesses, cause pain, and prevent a normal range of motion (ROM) [1,2]. Complete knee ankylosis in full extension, where no passive or active flexion is achievable, represents the most severe end of this spectrum and poses a unique technical challenge for arthroscopic intervention.

Standard arthroscopic portal placement relies on at least partial knee flexion, typically 70-90°, to open the tibiofemoral joint space and create a safe corridor for instrument insertion [3]. The anterolateral and anteromedial portals are sited at the level of the joint line on either side of the patellar tendon, and their safety depends on flexion in three respects: flexion opens the anterior tibiofemoral interval so that a trocar may pass above the meniscus without abutting articular cartilage; it displaces the infrapatellar fat pad posteriorly and inferiorly, away from the intended trajectory; and it allows the joint to be distended so that the entry point can be confirmed before instrumentation. When the knee is locked in full extension, none of these conditions is met. The anterior interval is closed, the fat pad lies compressed against the femoral condyles, and a trocar introduced at the joint line must therefore be advanced essentially blind towards articular cartilage. By contrast, the suprapatellar pouch lies proximal to the patella, between the quadriceps tendon and the anterior femoral cortex, and its position does not depend on knee flexion.

Kim and Kim [4], drawing on accumulated experience with more than 4,000 arthroscopies, described a philosophy of portal positioning in which portal location should adapt flexibly to the surgeon's need rather than follow a fixed template, and in which higher portal positions generally provide wider intra-articular visualisation; they specifically addressed the stiff knee. However, the application of this principle to complete post-traumatic ankylosis at 0° has not been described.

In this case report, we present a case of complete post-traumatic knee ankylosis in full extension treated with arthroscopic lysis of adhesions (LOA) using a superolateral portal-first approach. This work has been reported in line with the SCARE (Surgical CAse REport) 2023 criteria [5].

## Case presentation

Patient information and clinical findings

A 61-year-old woman (body mass index 21.37 kg/m²) presented to our institution three months after undergoing open reduction and internal fixation (ORIF) with bone grafting of the left proximal tibia at an outside provincial hospital. All time points in this report are expressed relative to this presentation, and postoperative time points relative to the day of arthroscopic arthrolysis. The specific fracture classification was not available from the referring institution. She reported progressive stiffness of the left knee that had not responded to physical therapy at the referring center. Her medical history was notable only for chronic hepatitis B virus carrier status. She had no history of diabetes, autoimmune disease, bleeding disorders, or prior knee surgery, was a non-smoker, and was a retired agricultural worker living with family support.

**Figure 1:**
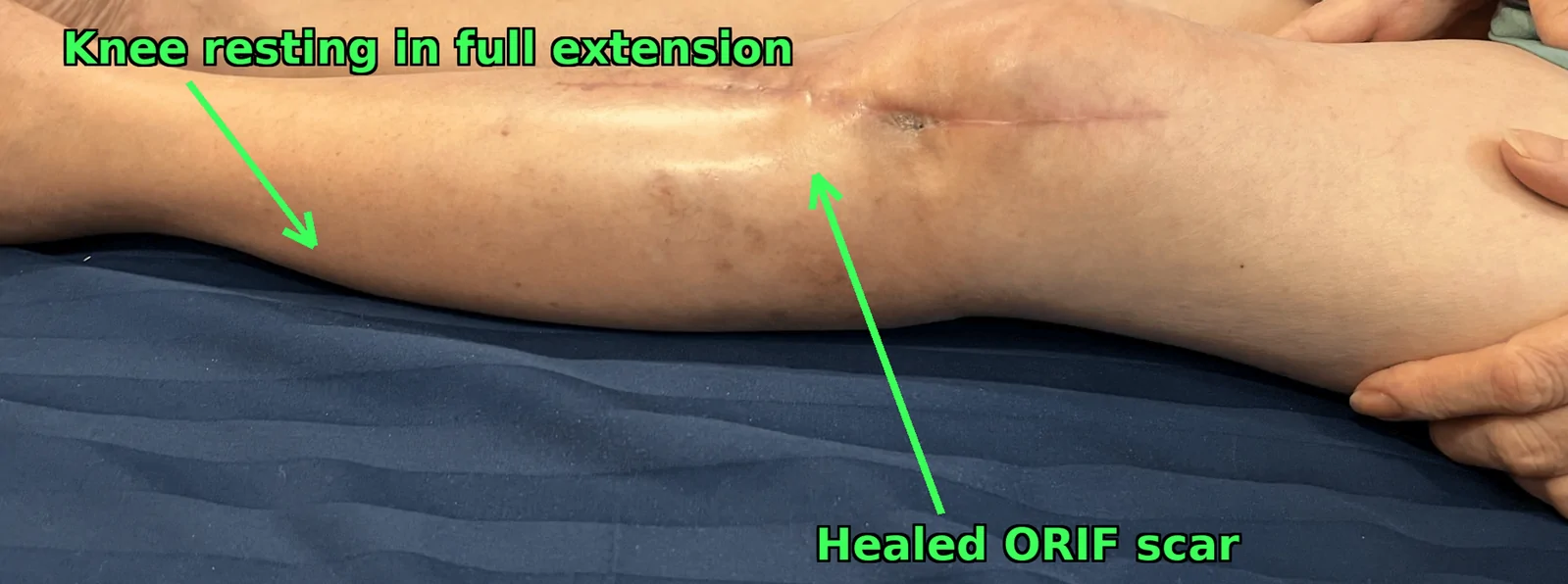
Preoperative appearance of the ankylosed left knee. The limb rests in full extension (arrow) with a well-healed longitudinal surgical scar over the proximal tibia from the prior open reduction and internal fixation with bone grafting (arrow). On examination, no passive or active flexion was achievable from this position.

On examination, she was alert and cooperative with stable vital signs (blood pressure 118/67 mmHg, heart rate 68 bpm, temperature 37.2°C). The left lower extremity showed a well-healed longitudinal surgical scar over the proximal tibia without erythema, warmth, or discharge. The left knee was completely rigid at 0° of extension and no passive or active flexion was achievable despite gentle sustained effort (Figure 1). Patellar mobility was severely restricted in both mediolateral and superoinferior directions, with diffuse periarticular firmness consistent with fibrosis. The contralateral right knee had full range of motion (ROM; 0-130°). Distal neurovascular status was intact and she ambulated with bilateral crutches. We did not record thigh circumference, manual muscle testing, or a formal test of quadriceps extensibility, and the extra-articular component of the contracture was therefore not quantified preoperatively; the implications of this omission are considered in the Discussion.

The presentation could not be graded using the existing classification systems. The four-type system of Shelbourne et al. [6] requires measurable extension and flexion deficits, with the most severe category (type 4) defined by an extension loss greater than 10° with 30° or more of flexion loss and patella infera. The international consensus statement of Kalson et al. [7] grades severity according to residual flexion (90-100°, 70-89°, or less than 70°) or extension deficit (5-10°, 11-20°, or greater than 20°). Our patient, with no achievable flexion whatsoever and full extension, fell outside both frameworks, exceeding the most severe defined category in each.

Diagnostic assessment

**Figure 2:**
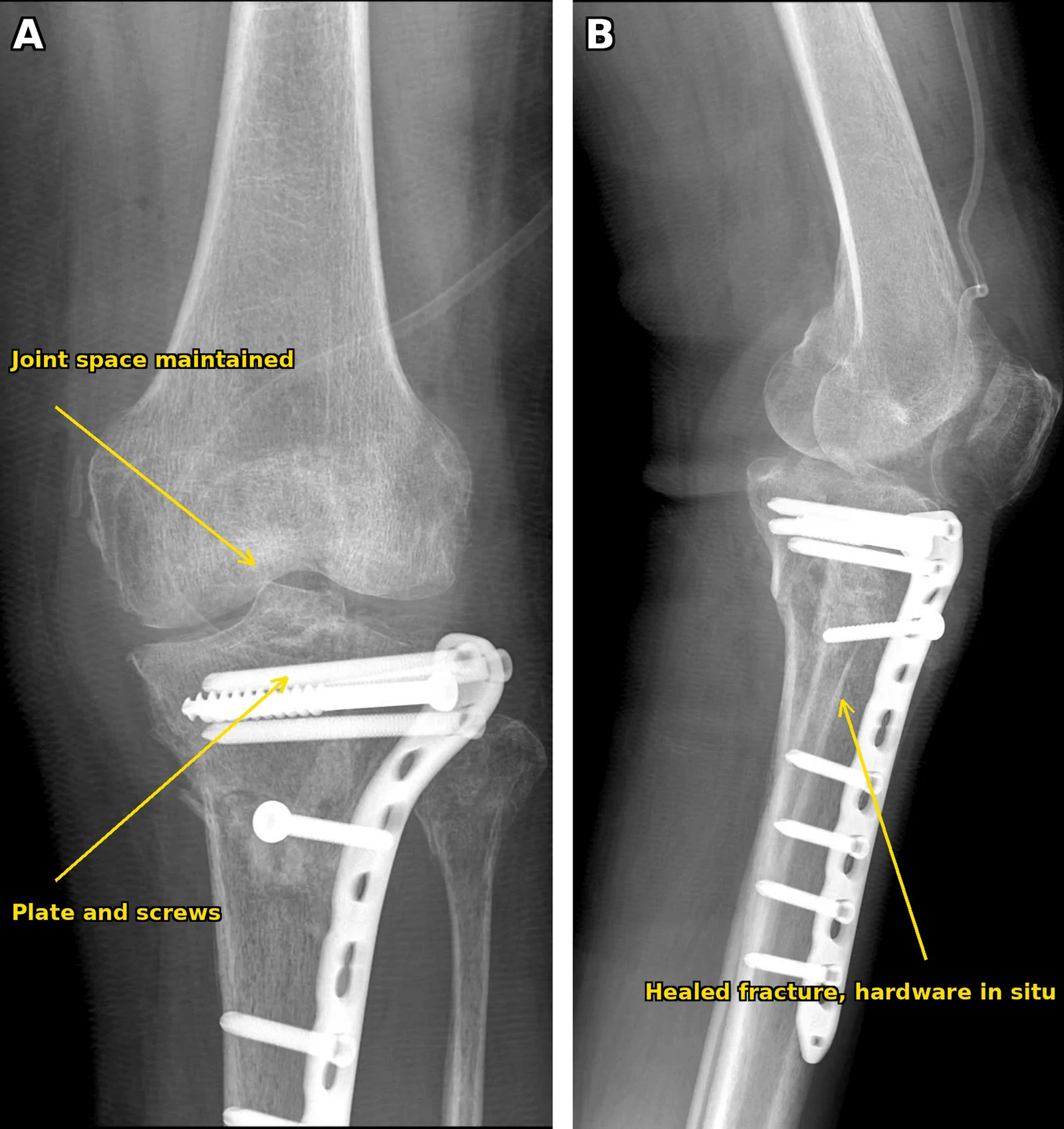
Preoperative radiographs of the left knee after index fixation. (A) Anteroposterior view showing the lateral buttress plate and screws in situ (arrow) with a maintained tibiofemoral joint space (arrow). (B) Lateral view showing the healed proximal tibial fracture with hardware in situ (arrow), without failure, loosening, or malposition. Diffuse periarticular osteopenia of the distal femur and proximal tibia is evident on both views.

Preoperative anteroposterior and lateral radiographs of the left knee demonstrated healed fracture fragments of the proximal tibia with a lateral buttress plate and screws in situ (Figure 2). No hardware failure, loosening, or malposition was identified, and the tibiofemoral joint space was maintained. Diffuse periarticular osteopenia of the distal femur and proximal tibia was evident, consistent with prolonged disuse. Ultrasonography revealed a small joint effusion, a thickened Hoffa fat pad, and periarticular soft tissue changes suggestive of diffuse fibrosis. Magnetic resonance imaging was not performed owing to anticipated metallic artifact. Laboratory workup including complete blood count, C-reactive protein, erythrocyte sedimentation rate, and hepatic function tests was within normal limits, and hepatitis B viral load was undetectable. The patient was diagnosed with post-surgical arthrofibrosis of the left knee after three months of failed conservative management.

Rationale for the access strategy

Manipulation under anesthesia (MUA) as an isolated procedure was considered and rejected for three reasons. First, MUA relies on progressively increasing an existing arc of motion; with the knee ankylosed at 0°, there was no arc through which controlled force could be applied. Second, the clinical and ultrasonographic findings indicated dense fibrous obliteration of all joint recesses, which isolated manipulation could only disrupt indiscriminately rather than release selectively. Third, three months of non-weight-bearing immobilisation raised concern for disuse osteopenia, and forceful manipulation of a rigid joint in compromised bone carries a recognised risk of iatrogenic supracondylar fracture and extensor mechanism disruption [8]. Arthroscopic release under direct vision, followed by gentle incremental manipulation once the adhesions had been divided, was therefore selected as the safer sequence.

**Figure 3:**
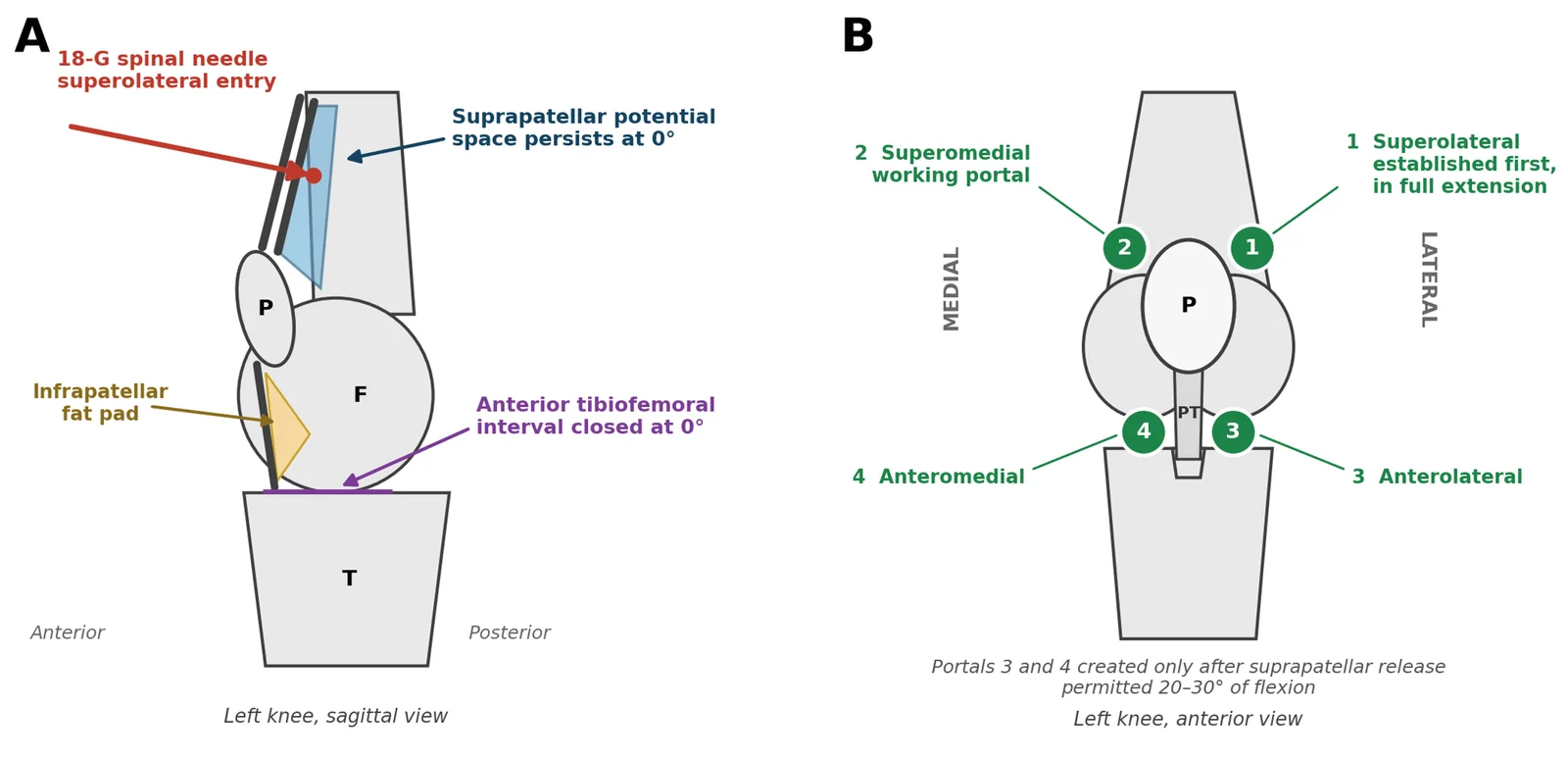
Schematic of the superolateral portal-first approach. (A) Sagittal view of the left knee at 0°, anterior to the left. The anterior tibiofemoral interval is closed and the infrapatellar fat pad is compressed, so a trocar placed at the joint line would be advanced blind towards articular cartilage. The suprapatellar potential space (shaded) lies between the quadriceps tendon and the anterior femoral cortex and persists independently of flexion; it is entered with an 18-gauge spinal needle directed from a superolateral position parallel to the anterior femoral cortex. P, patella; F, femur; T, tibia. (B) Anterior view of the left knee showing the sequence of portal creation. The superolateral portal (1) is established first with the knee in full extension, followed by the superomedial working portal (2); the anterolateral (3) and anteromedial (4) portals lie at the joint line on either side of the patellar tendon (PT) and were created only after suprapatellar release had permitted 20-30° of flexion. This schematic is original and was created by the authors.

We also considered establishing a conventional anterolateral portal and creating a working space gradually under direct arthroscopic vision, as is often possible in the moderately stiff knee. We rejected this because at 0°, the anterior interval is closed and the obliterated fat pad offers no distensible plane: the initial trocar pass would necessarily have been blind and directed towards the femoral condyle, with attendant risk of iatrogenic chondral injury or of creating a false extra-articular passage, and poor distension would have limited visualis

ation from the outset. The superolateral route avoids both problems, since it enters a space whose existence does not depend on flexion and whose trajectory lies proximal to the tibiofemoral articular surfaces (Figure 3).

Surgical technique

The patient was placed supine under spinal anesthesia (bupivacaine 0.5%, 12.5 mg intrathecally). A pneumatic tourniquet (Zimmer Biomet, Warsaw, IN, USA) was applied to the proximal left thigh and inflated to 300 mmHg. Examination under anesthesia confirmed complete ankylosis at 0° with no achievable passive flexion. Prophylactic intravenous cefazolin 2 g was administered.

**Figure 4:**
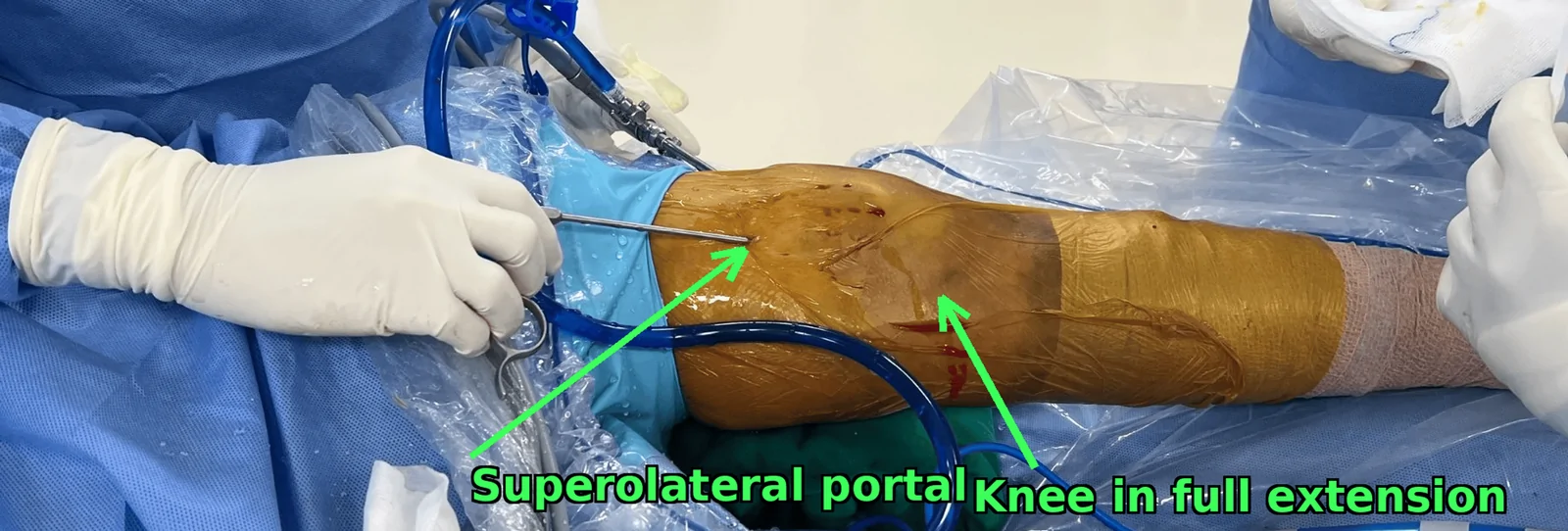
Superolateral portal established with the knee in full extension. The arthroscope has been introduced through the superolateral portal, directed at the suprapatellar potential space beneath the superior patellar pole (arrows). No knee flexion was required to establish this portal.

**Figure 5:**
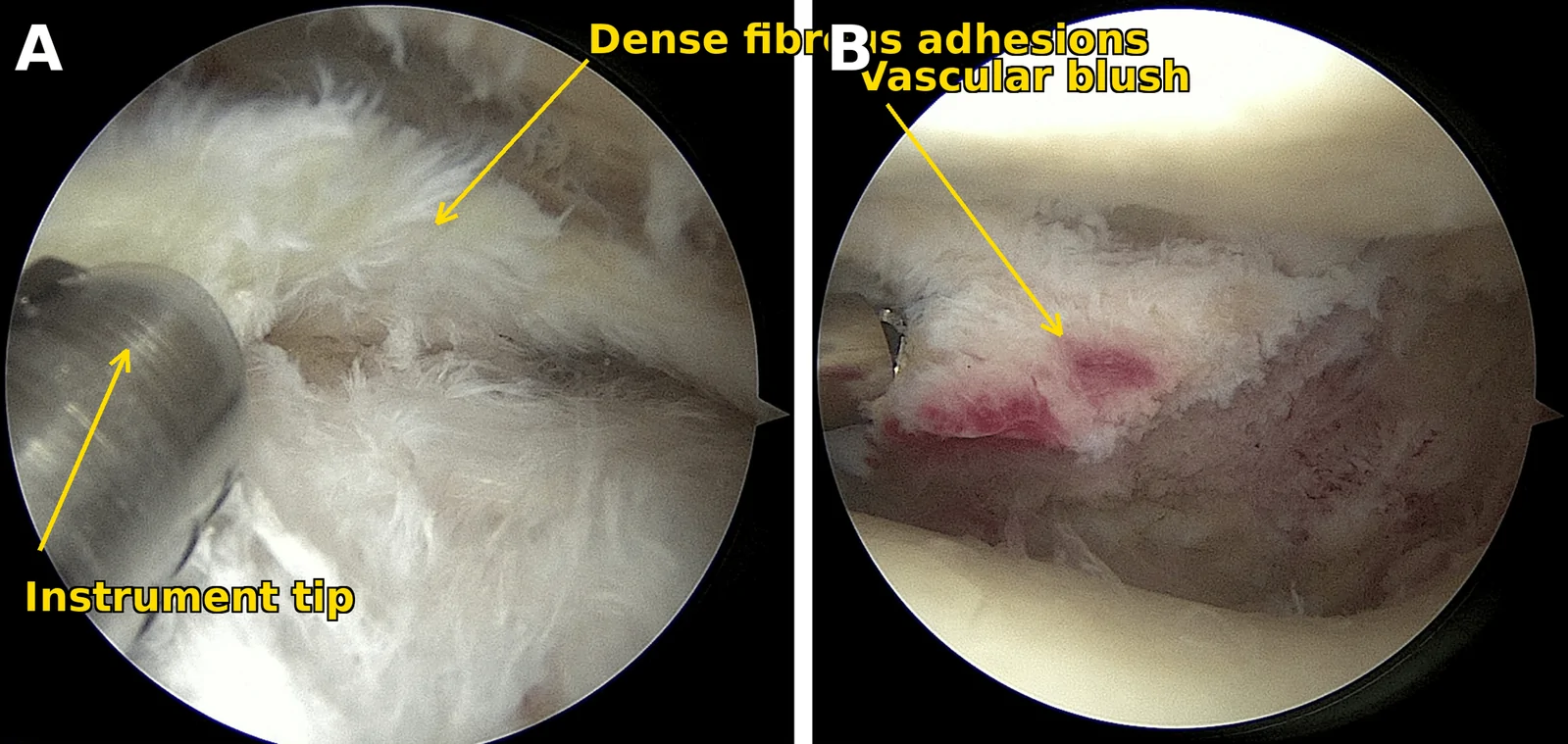
Arthroscopic appearance of the suprapatellar pouch on initial inspection. (A) Dense, organised, curtain-like fibrous adhesions with radiating strands bridging the quadriceps tendon to the anterior femoral cortex (arrow); a blunt instrument tip is visible at the left of the field for scale and retraction (arrow). (B) Prominent vascular blush within the fibrous mass (arrow), indicating an actively vascularised rather than a quiescent fibrotic process.

Step 1: Superolateral portal establishment in full extension. Because the tibiofemoral joint space was inaccessible with the knee locked in full extension, standard anterolateral portal placement was not feasible. The superolateral corner of the patella was palpated and marked. An 18-gauge spinal needle was introduced approximately one fingerbreadth proximal and lateral to this point and angled slightly distally and medially so as to run parallel to the anterior femoral cortex and pass beneath the articular surface of the superior patellar pole [4] (Figure 3A). Intra-articular position was confirmed by loss of resistance, by the ease with which 30 mL of normal saline could be injected without periarticular soft-tissue swelling, and by spontaneous backflow on disconnecting the syringe. Had backflow not been obtained, our intention was to reposition the needle more proximally along the anterior femoral cortex before making any skin incision, and to proceed to a mini-open superolateral arthrotomy to enter the pouch under direct vision if the space still could not be entered; neither manoeuvre proved necessary. Only after the space had been confirmed by distension was a superolateral portal created with a No. 11 blade (Figure 4). A 4.0 mm 30° arthroscope (Stryker, Kalamazoo, MI, USA) was introduced and confirmed dense, diffuse, curtain-like adhesions within the suprapatellar pouch (Figure 5).

**Figure 6:**
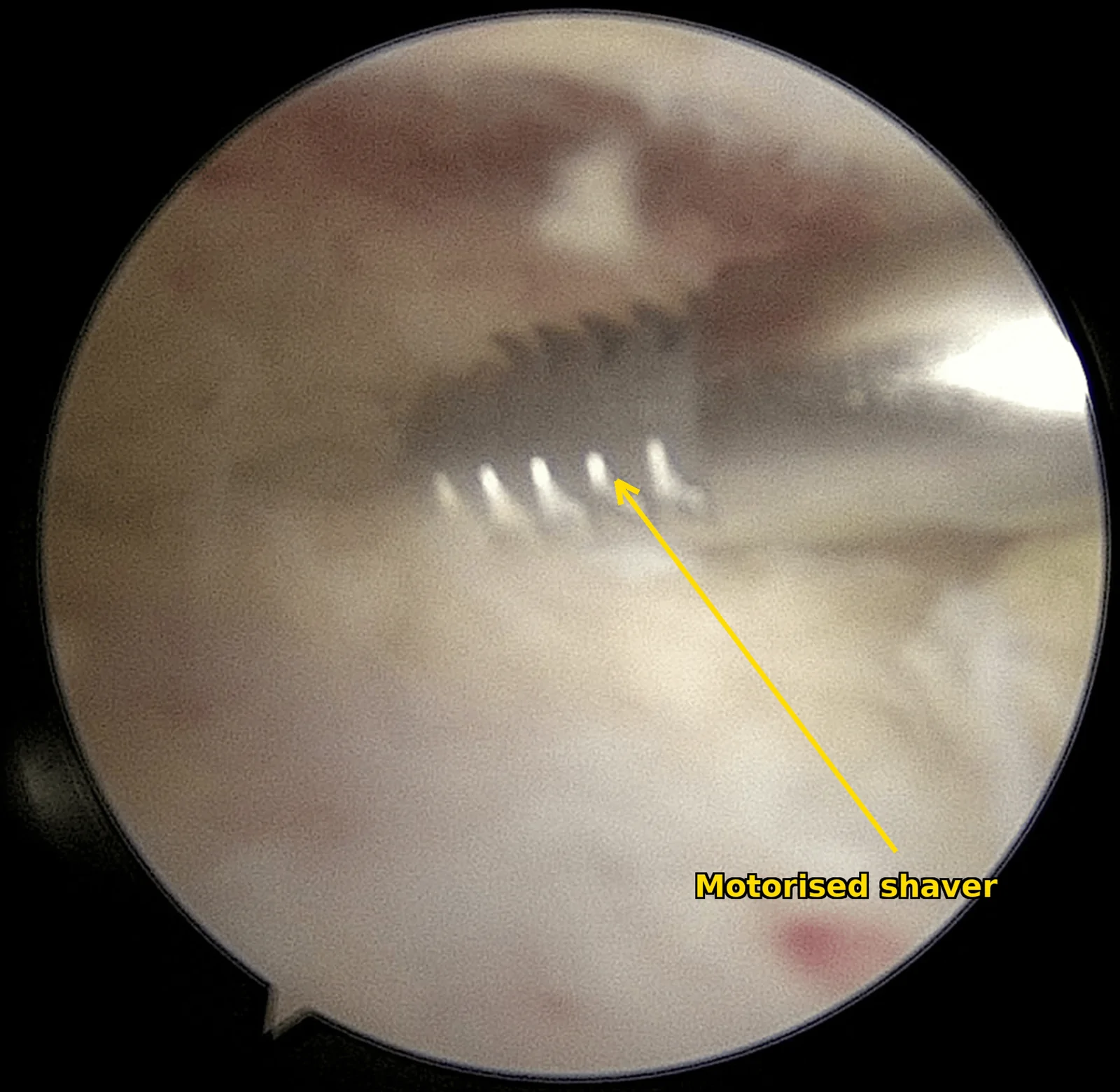
Arthroscopic debridement of the suprapatellar adhesions. The motorised shaver is engaged in the dense fibrous tissue (arrow). Debridement proceeded systematically while preserving the integrity of the overlying quadriceps tendon.

**Figure 7:**
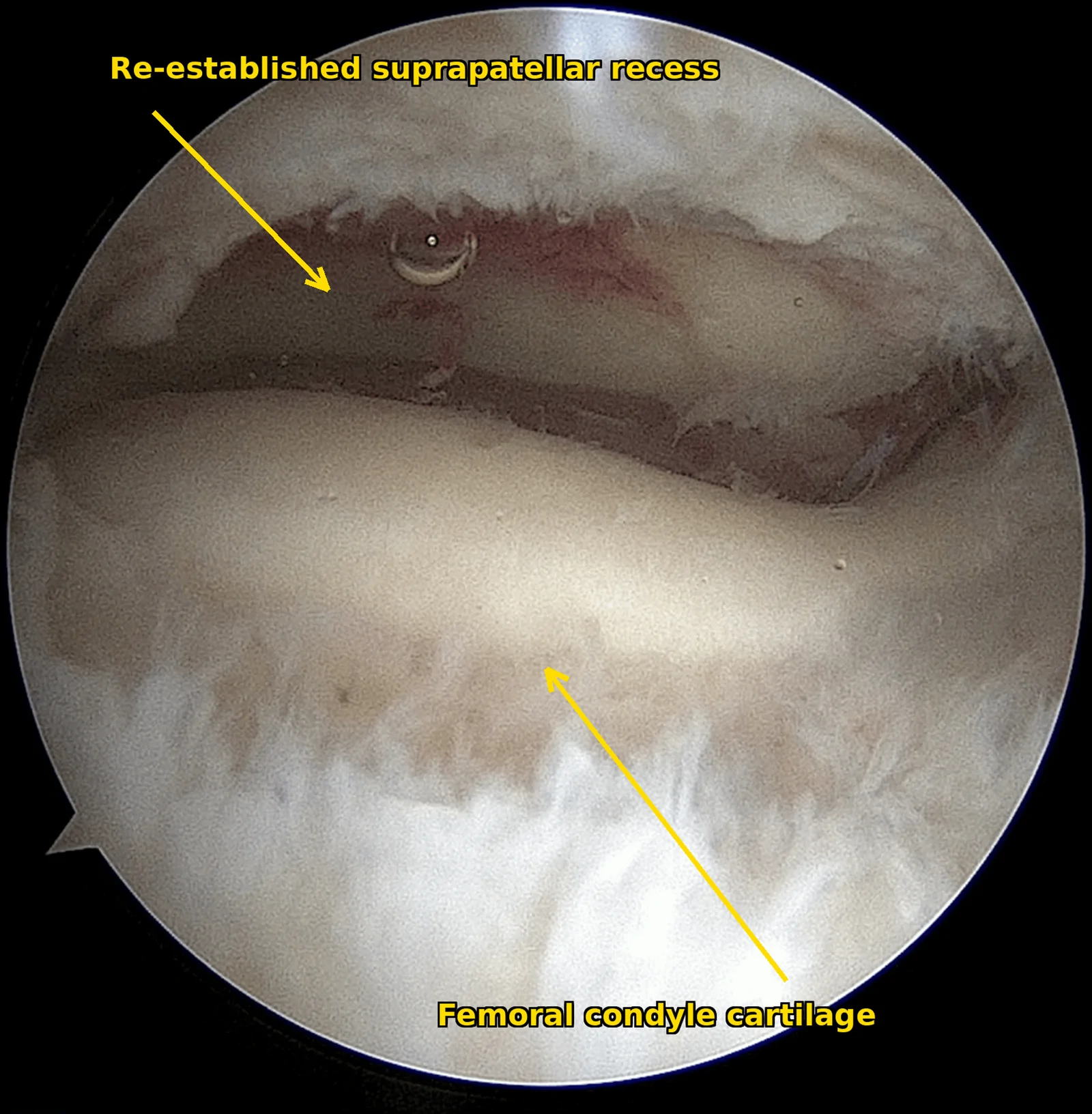
Arthroscopic appearance after debridement. The suprapatellar recess has been re-established (arrow) with the smooth articular surface of the femoral condyle exposed (arrow). The cartilage is relatively preserved, corresponding to Outerbridge grade I-II in the weight-bearing zones.

Step 2: Suprapatellar pouch debridement. A superomedial portal was established under direct arthroscopic visualisation and a motorised shaver (4.5 mm Full Radius Resector; Stryker) was introduced through it. Systematic debridement of the dense adhesions bridging the quadriceps tendon to the anterior femoral cortex was performed, with meticulous resection of the fibrous tissue filling the suprapatellar pouch while preserving the integrity of the quadriceps tendon (Figure 6). Re-establishment of the suprapatellar recess after resection of the overlying adhesions is shown in Figure 7. Reduced bone quality was noted intraoperatively (cortical thinning on probing), consistent with disuse osteopenia. Tissue specimens were sent for microbiological culture and histopathological examination.

Step 3: Progressive portal creation and peripatellar release. Following debridement of the suprapatellar adhesions, sufficient mobility was achieved to allow initial gentle flexion of approximately 20-30°. The adhesive tissue encasing the patella was addressed by releasing the medial and lateral peripatellar adhesions using a radiofrequency ablation device (VAPR; DePuy Mitek, Raynham, MA, USA). With progressive mobility, standard anterolateral and anteromedial portals were then established (Figure 3B).

Step 4: Infrapatellar and intercondylar debridement. Through the anterior portals, dense fibrous tissue within the infrapatellar fat pad, pretibial recess, intercondylar notch, and medial and lateral gutters was systematically debrided. The radiofrequency device was used for the resection of thicker adhesive bands and for hemostasis. The retained fixation hardware was identified in situ after clearance of the surrounding fibrous tissue. Articular cartilage of the femoral condyles and tibial plateau was Outerbridge grade I-II in the weight-bearing zones. The posterior compartment was then inspected arthroscopically through the intercondylar notch; no significant posterior capsular or posterior recess adhesions were identified and no posterior release was performed.

Step 5: Gentle incremental manipulation. After comprehensive debridement, the knee was manipulated under direct arthroscopic visualisation. Flexion was increased in stages to 40°, then 60° with gentle sustained pressure, and finally 90°. Gradual steady force was applied with reassessment between each increment and with continuous monitoring for crepitus or sudden give; no crepitus was detected. Given the compromised bone quality, a predetermined threshold was set to abandon further manipulation in favour of staged intervention had resistance failed to yield to steady pressure.

Step 6: Closure. A closed-suction drain (Hemovac; Zimmer Biomet) was placed through the anteromedial portal and portal incisions were closed with 4-0 nylon interrupted sutures. A hinged knee brace (DonJoy, Vista, CA, USA) was applied, set to allow 0-90° of motion. Total operative time was 95 minutes and intraoperative blood loss was approximately 50 mL. No intraoperative complications occurred.

Postoperative care

Immediate postoperative management included multimodal analgesia with oral paracetamol 500 mg three times daily, intravenous nefopam 30 mg as needed, and zolpidem 7.5 mg at night. Continuous passive motion (CPM; Kinetec Spectra, Tournes, France) was initiated within six hours, starting at 0-40° at one cycle per minute and advanced by 5-10° daily as tolerated, applied for six to eight hours daily. Supervised physiotherapy was conducted twice daily and consisted of quadriceps isometric strengthening, straight-leg raises, and active-assisted ROM exercises. Weight-bearing was permitted as tolerated with crutches. Enoxaparin 40 mg subcutaneously daily was given for thromboprophylaxis. The drain was removed at 48 hours with an output of 120 mL of serous fluid. Intraoperative tissue cultures were negative at five days, and histopathological examination confirmed dense fibrous tissue with a chronic inflammatory infiltrate consistent with arthrofibrosis.

Outcome and follow-up

At discharge on postoperative day 8, the patient demonstrated active knee flexion of approximately 50°, passive flexion of 85° on CPM, full active extension (0°) and clean dry portal wounds without signs of infection. She was ambulatory with crutches and able to perform activities of daily living with minimal assistance. She was discharged with a structured home exercise program including daily CPM use, active-assisted ROM exercises three times daily, quadriceps strengthening, and progressive weight-bearing, with the hinged brace worn between exercise sessions.

**Figure 8:**
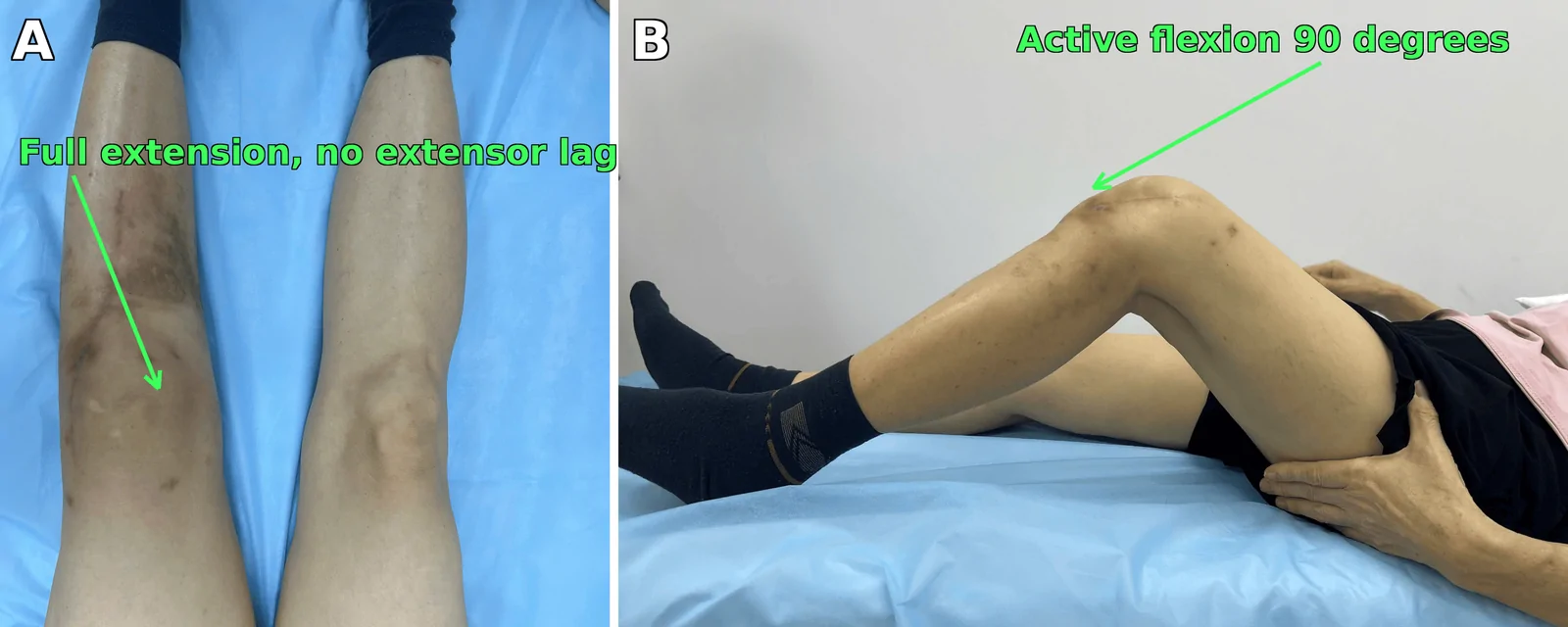
Clinical photographs at one-month follow-up. (A) Both lower limbs in full extension; the operated left knee (arrow) maintains full active extension (0°) without extensor lag, symmetrical with the contralateral right knee, and the surgical scar and all portal sites are well healed. (B) Active knee flexion of 90° measured by goniometry (arrow), compared with a preoperative value of 0°.

At one-month follow-up, the surgical scar and all portal sites had healed without complication, and there was no effusion, erythema, or warmth. Full active extension of 0° was maintained, symmetrical with the contralateral limb and without extensor lag (Figure 8A). Active knee flexion measured 90° by goniometry (Figure 8B), compared with a preoperative value of 0°. Notably, active flexion had progressed from 50° at discharge to 90° at one month, matching the arc achieved passively under anesthesia and indicating both retention of the surgical release and recovery of active quadriceps and hamstring control. No complications, including infection, iatrogenic fracture, extensor mechanism disruption, or recurrent stiffness, were observed. The retained fixation hardware remained in situ. This is the most recent time point at which the patient has been assessed, and no validated patient-reported outcome measure was recorded at this or any earlier visit.

## Discussion

This case demonstrates that a superolateral portal-first approach permitted safe initial arthroscopic access in a knee ankylosed at 0°, in which conventional anterior portal placement was not feasible. The case is notable for the severity of the initial presentation and for the achievement at one month of an active 90° arc equal to the passive arc obtained intraoperatively.

The technique is not novel in isolation. Kim and Kim [4] described the principle of high portal placement for the stiff knee in 2001, and a recent technical report has detailed comprehensive arthroscopic arthrolysis including suprapatellar debridement [9]. Our contribution is narrower: the application of this principle to complete post-traumatic ankylosis at 0°, a presentation that falls outside the range of severity addressed in the existing literature and outside all published classification systems. We make no claim that this sequence is superior to a conventional anterior approach in knees that retain some flexion; our contention is confined to the situation in which the knee cannot be flexed at all.

Extra-articular contribution to stiffness

A point deserving emphasis is that complete ankylosis in full extension is an atypical pattern for isolated intra-articular arthrofibrosis. Adhesions confined to the joint recesses characteristically restrict flexion while preserving some arc; total loss of flexion with preserved full extension raises the possibility of a concomitant extra-articular component, in particular quadriceps contracture or adherence of the extensor mechanism to the underlying fracture callus. This distinction is clinically consequential, because predominantly extra-articular contracture is the classical indication for quadricepsplasty rather than arthroscopic release [1]. In our case, the intraoperative finding that suprapatellar debridement alone restored 20-30° of flexion, and that comprehensive intra-articular release permitted 90° without any extra-articular procedure, indicates that the dominant pathology was intra-articular. The subsequent recovery of active flexion to 90° with full active extension and no extensor lag at one month further argues against a clinically dominant extensor mechanism contracture, since a significantly contracted or scarred quadriceps would be expected to limit active flexion disproportionately.

These are nonetheless inferences drawn from the intraoperative and postoperative course rather than from direct preoperative measurement. Because we did not record thigh circumference, manual muscle testing, or a formal test of quadriceps extensibility such as the prone rectus femoris (Ely) test, a reader cannot independently verify the relative contributions of intra-articular and extra-articular contracture in this patient and cannot use our description to match a future patient on that basis. We regard this as the principal weakness of our preoperative assessment and recommend that these simple measurements be documented prospectively whenever this presentation is encountered since a knee that fails to gain flexion after thorough intra-articular release is unlikely to be salvaged by further arthroscopic work and should prompt consideration of an extra-articular procedure.

Treatment algorithm and rationale

Management follows a stepwise approach, beginning with aggressive physiotherapy, serial static splinting, multimodal analgesia, and anti-inflammatory medication [1]. When conservative measures fail, MUA is typically attempted. In a systematic review of the stiff total knee arthroplasty, Fitzsimmons et al. [10] found that gains in ROM after MUA and after arthroscopy with or without MUA were similar, that open arthrolysis produced inferior gains, and that MUA was more successful when performed early, although it could remain effective when performed late; the numbers of clinically important complications after MUA and after arthroscopy were similar.

A systematic review by Fackler et al. [11] of eight studies comprising 240 patients found that arthroscopic LOA with MUA produced a mean improvement of 41.6° in arc of motion, with clinically significant improvements in International Knee Documentation Committee, Western Ontario and McMaster Universities Osteoarthritis Index, and Knee injury and Osteoarthritis Outcome Score across all applicable studies. A single complication, a synovial fistula that resolved without intervention, occurred among the 240 patients. Arthroscopic LOA offers direct visualisation of intra-articular pathology, targeted resection across multiple compartments, reduced surgical trauma enabling immediate postoperative mobilisation, and lower risk of wound complications [9,12,13]. In our patient, the arthroscopic approach permitted initiation of CPM within six hours with minimal wound morbidity, which we consider critical to maintaining the intraoperative gain.

Portal strategy in the completely ankylosed knee

The fundamental challenge is gaining initial arthroscopic access to a joint that cannot be flexed. Standard anterolateral and anteromedial portals are created with the knee flexed, which opens the tibiofemoral articulation and allows safe trocar passage; accurate portal placement depends on the mastery of surface anatomy and is critical to both diagnostic and operative arthroscopy [3]. When the knee is locked in extension, this corridor is compressed and effectively inaccessible. The solution lies in the observation that the suprapatellar pouch, while typically obliterated by adhesions, often retains a small potential space just beneath the superior patellar pole [4]. Kylies et al. [9] described a comprehensive arthroscopic arthrolysis technique including systematic debridement of the suprapatellar pouch, gutters, infrapatellar region, intercondylar notch, and posterior recesses.

This proximal-to-distal approach offers several advantages. It bypasses the inaccessible tibiofemoral joint for initial access; debriding the suprapatellar adhesions first yields sufficient initial flexion to allow standard anterior portal placement; progressive portal creation minimises the risk of articular cartilage injury that would occur if anterior portals were forced through a rigid joint; and because the needle and arthroscope pass proximal to the tibiofemoral articular surfaces, a misdirected initial pass cannot scuff them. The posterior compartment was inspected arthroscopically through the intercondylar notch once the anterior release was complete. No significant posterior capsular or posterior recess adhesions were identified and no posterior release was therefore performed; this contrasts with the flexion-contracture presentations for which posterior capsular release is described [9,13] and illustrates that the extent of release should follow both the direction of the deficit and the findings on inspection. Direct inspection matters here, because preserved extension alone would not exclude posterior pathology: posterior adhesions restrict flexion while leaving extension unaffected. Technical pearls and pitfalls are summarised in Table 1.

**Table 1:** Technical pearls and pitfalls of the superolateral portal-first approach in the completely ankylosed knee ROM: Range of motion; CPM: continuous passive motion. This table is original and was created by the authors.

Pearls	Pitfalls
The suprapatellar space beneath the superior patellar pole is the most reliable initial entry point in a completely ankylosed knee, as it typically retains a small residual potential space.	Avoid forceful manipulation in osteopenic bone; apply gradual sustained pressure in increments, reassess between each, and abandon if resistance does not yield.
Establish the superolateral portal first in full extension; confirm intra-articular position by saline distension and backflow before creating any skin incision.	Prolonged arthroscopy increases the risk of fluid extravasation and compartment syndrome; monitor limb compartments throughout and limit operative time.
Create standard anterior portals only after achieving 20-30° of flexion through suprapatellar adhesiolysis (progressive portal creation).	Retained fracture fixation hardware may independently limit ROM; consider removal once bone healing is confirmed.
Use radiofrequency ablation for both tissue resection and hemostasis in the highly vascular adhesive tissue.	Failure to gain flexion after thorough intra-articular release suggests a dominant extra-articular contracture that arthroscopy will not address.
Initiate continuous passive motion within 24 hours and advance ROM targets 5-10° daily to prevent re-adhesion.	Inadequate postoperative rehabilitation is the most common cause of recurrent stiffness; ensure compliance with CPM and physiotherapy.

Timing and bone quality

Our timing of intervention at three months after the index procedure is supported by the evidence. Eggeling et al. [14] studied 123 patients undergoing arthroscopic arthrolysis and reported a mean ROM increase from 73.9° to 131.4° (p<0.001). Stratified by timing, normal ROM was restored in 76% of patients treated within three months, 68% of those treated between three and six months, and 41.5% of those treated after six months (p=0.005), with postoperative flexion deficits of 3.9°, 4.2°, and 16.6°, respectively (p<0.001). Fahlbusch et al. [15] similarly reported a mean interval from index surgery to arthroscopic LOA of 27.7±12.8 weeks with a mean ROM gain of 48.0°±10.6°.

The observation of reduced bone quality warrants emphasis and was supported both radiographically, by the diffuse periarticular osteopenia visible on the preoperative films (Figure 2), and intraoperatively by cortical thinning on probing. Disuse osteopenia is a recognised consequence of prolonged immobilisation and has direct implications for the safety of manipulation, since iatrogenic fracture is a known although uncommon complication of both MUA and arthroscopic LOA [8]. Achieving 90° of flexion in a single sitting in a knee with compromised bone quality represents, in our view, the upper limit of what should be attempted. We emphasise that force should be applied gradually and in increments with reassessment between each, and that manipulation should be abandoned in favour of staged intervention if resistance does not yield to steady sustained pressure. We would caution against reproducing the magnitude of correction reported here as a target in itself.

Why flexion did not exceed 90° deserves comment. The endpoint of 90° was a deliberate intraoperative decision rather than a predetermined therapeutic target: manipulation was advanced in increments and was stopped at 90° because the combination of radiographic and palpable osteopenia made further force appear imprudent, and the hinged brace was set to 0-90° accordingly. Two factors may nonetheless have contributed to the residual deficit. The retained proximal tibial hardware may have acted as a mechanical tether and can independently promote scar formation and an unquantified extra-articular component of the contracture may have limited excursion of the extensor mechanism. Residual posterior compartment pathology is unlikely to have been responsible, since the posterior compartment was inspected directly and no significant adhesions were identified. Should flexion plateau or regress with longer follow-up, a staged approach would be reasonable: implant removal combined with repeat arthroscopic arthrolysis, with consideration of an extra-articular procedure if a further intra-articular release again fails to produce gain. We did not pursue greater correction at the index procedure because the risk of iatrogenic fracture in osteopenic bone outweighed the incremental benefit of a larger arc obtained in a single sitting.

Outcomes in context

Published outcomes of arthroscopic arthrolysis are generally favorable. In the earliest series, Sprague et al. [16] treated 24 patients with severely limited knee motion following open knee surgery and reported a mean increase in passive flexion from 70° to 115°. Kim and Joo [17] evaluated 68 patients and found that 61 (89.7%) obtained a mean ROM increase of 48.6° while seven (10.3%) showed no increase, with duration of disease being the significant prognostic factor. Abdelghafour et al. [18] prospectively studied 30 patients with post-traumatic knee arthrofibrosis and reported ROM improving from 75.0°±10.9° to 119.8°±10.4° and Lysholm score from 56.9±2.6 to 85.3±3.5 at a mean follow-up of 6.7 months (both p<0.001). For post-arthroplasty stiffness, Jerosch and Aldawoudy [12] reported a mean flexion of 119° at the end of arthroscopy in 32 patients, which had settled to 97° at final follow-up, and Yayac et al. [19] reported improvements of 51.2° in flexion, 8.9° in extension, and 60° in total ROM in 103 patients at a mean follow-up of 1.7 years.

Three observations follow. First, most published series include patients who retain substantial residual ROM preoperatively; the mean preoperative ROM was 75° in the series of Abdelghafour et al. [18] and 73.9° in that of Eggeling et al. [14]. Complete ankylosis at 0° is rarely reported in the context of arthroscopic treatment, which both limits direct comparison and, we would argue, justifies documenting this presentation. Second, these series report Lysholm or equivalent scores as their functional endpoint, whereas we report range of motion alone; our result therefore cannot be placed alongside them on any functional axis. Third, the observation of Jerosch and Aldawoudy [12] that flexion settled from 119° intraoperatively to 97° at follow-up illustrates that a proportion of the intraoperative gain is characteristically lost. Our patient had converted the intraoperative passive arc into an equivalent active arc by one month, which we interpret as a favourable early trajectory, but this interval is short and the course beyond it is unknown.

Limitations

This report has important limitations, several of which bear directly on how far its conclusions can be taken.

The most significant is the duration of follow-up. Observation extends only to one month after arthrolysis. Arthrofibrosis characteristically recurs over a longer interval, and the comparator series cited above report follow-up of 6.7 months to 1.7 years; the natural history of this condition is such that a one-month assessment falls well within the window in which recurrence would not yet be apparent. Our result must therefore be read as an early technical and clinical observation and not as evidence of clinical success, sustained benefit, or durability. We do not regard one month as sufficient to demonstrate that the arthrolysis has succeeded in any lasting sense.

Second, no validated patient-reported outcome measure such as the Lysholm or Oxford Knee Score was recorded at any time point. ROM is not a measure of function: a knee may achieve a 90° arc while remaining painful, unstable in the patient's perception, or limited in the activities that matter to her. The absence of such an instrument prevents any objective functional comparison between this patient and the existing literature, in which these scores are the primary endpoint, and it limits the report to a description of motion regained.

Third, the proximal tibial fixation hardware was not removed. Retained hardware around the knee can itself promote scar formation and may contribute mechanically to restriction. We are therefore unable to determine with certainty whether the ankylosis in this patient was attributable purely to post-surgical arthrofibrosis or was compounded by the presence of the implant, and this uncertainty cannot be resolved retrospectively; it would require reassessment of ROM after implant removal. Removal remains under consideration with the referring surgeon.

Fourth, as discussed above, the extra-articular component of the contracture was not quantified preoperatively. Fifth, the single-case design limits generalisability and the absence of a control group precludes comparative analysis; in particular, this report does not establish that a superolateral portal-first approach is superior or preferable to conventional anterior portal techniques, and no such comparison was attempted. Sixth, the original fracture classification and surgical details from the referring institution were incompletely documented. Seventh, intraoperative photographic documentation of the flexion arc obtained is not presented; the intraoperative range of motion was recorded contemporaneously in the operative record. Eighth, preoperative magnetic resonance imaging or computed tomography was not obtained owing to metallic artifact. Finally, the experience is that of a single surgeon at a single institution. We intend to report longer-term follow-up incorporating validated outcome measures in due course.

## Conclusions

Arthroscopic lysis of adhesions using a superolateral portal-first technique provided safe initial joint access in a knee ankylosed at 0°, in which conventional anterior portal placement was not feasible. The technique exploits the suprapatellar potential space, which persists independently of knee flexion, and employs systematic proximal-to-distal debridement with progressive portal creation. In this patient, an intraoperative arc of 90° was obtained and was matched by active flexion of 90° with full active extension at one month. We do not claim that this approach is superior or preferable to conventional anterior portal techniques, and a single case observed for one month cannot establish clinical success or durability, since arthrofibrosis characteristically recurs over a longer interval. The technique may nonetheless represent a useful option in selected cases of severe arthrofibrosis in which the knee cannot be flexed sufficiently to permit standard portal placement. Longer follow-up incorporating validated patient-reported outcome measures, and comparison with alternative access strategies, are required before any broader recommendation can be made.
